# Cathepsin B in cardiovascular disease: Underlying mechanisms and therapeutic strategies

**DOI:** 10.1111/jcmm.70064

**Published:** 2024-09-09

**Authors:** Zhulan Cai, Shunyao Xu, Chen Liu

**Affiliations:** ^1^ Department of Cardiology Peking University Third Hospital Beijing P.R. China; ^2^ Department of Critical Care Medicine, Shenzhen People's Hospital Second Clinical Medical College of Jinan University, First Affiliated Hospital of Southern University of Science and Technology Shenzhen P.R. China; ^3^ Department of Geriatrics The First Affiliated Hospital of Shenzhen University, Shenzhen Second People's Hospital Shenzhen P.R. China

**Keywords:** atherosclerosis, cardiomyopathy, cardiovascular diseases, cathepsin B, heart failure, hypertension, myocardial infarction, therapeutic strategies

## Abstract

Cathepsin B (CTSB) is a member of the cysteine protease family, primarily responsible for degrading unnecessary organelles and proteins within the acidic milieu of lysosomes to facilitate recycling. Recent research has revealed that CTSB plays a multifaceted role beyond its function as a proteolytic enzyme in lysosomes. Importantly, recent data suggest that CTSB has significant impacts on different cardiac pathological conditions, such as atherosclerosis (AS), myocardial infarction, hypertension, heart failure and cardiomyopathy. Especially in the context of AS, preclinical models and clinical sample imaging data indicate that the cathepsin activity‐based probe can reliably image CTSB activity in foam cells and atherosclerotic plaques; concurrently, it allows synchronous diagnostic and therapeutic interventions. However, our knowledge of CTSB in cardiovascular disease is still in the early stage. This paper aims to provide a comprehensive review of the significance of CTSB in cardiovascular physiology and pathology, with the objective of laying a theoretical groundwork for the development of drugs targeting CTSB.

## 1 INTRODUCTION

Cardiovascular diseases persist as a major threat to human health. Cathepsin B (CTSB), a ubiquitous lysosomal cysteine protease, participates in a multitude of physiological processes and plays a pivotal role in the progression of diverse pathological conditions. Historically, the study of CTSB has mainly focused on the field of oncology.^1^ Nevertheless, a growing body of recent research has begun to unveil the intricate involvement of CTSB in cardiovascular diseases. Yet, the exact mechanisms by which it contributes to their aetiology are still not fully understood. In this comprehensive review, we provide an in‐depth understanding of the role of CTSB in a range of cardiovascular diseases, elucidate its underlying mechanisms of action, and explore its practical applications within the context of disease progression.

## 2 THE CATHEPSIN FAMILY

Cathepsins, also referred to as peptidases or proteolytic enzymes, are extensively expressed in diverse organisms and constitute a group of lysosomal digestive enzymes crucial for preserving cellular homeostasis.^2^, ^3^ In humans, 15 types of cathepsins have been identified and categorized into three main classes according to their catalytic domains: aspartate, serine and cysteine.^3^ Aspartic cathepsins consist of cathepsin D and cathepsin E, while serine cathepsins include cathepsin A and cathepsin G. Cysteine cathepsins are categorized as cysteine proteases of the papain family (C1 family) due to their homology with papain, a protease presents in papaya plants. The C1 family, the largest family of tissue proteases, comprises 11 members: cathepsins B, C, F, H, K, L, V, O, S, W and Z.^4^

Nonetheless, cathepsins are not exclusively confined to lysosomes; their localization can shift under diverse circumstances. For instance, they may relocate to the nucleus to participate in histone cleavage and regulate gene expression, translocate to the cell surface for secretion into the extracellular matrix (ECM) for various functions, or enter the circulation and be detected in the serum or plasma.^5^, ^6^ Recent research advancements have revealed that the tissue cell specificity or subcellular localization of cathepsin dictates their physiological functions, leading to their involvement in diverse physiological and pathological processes across various cell types and organelles, resulting in distinct outcomes.^7^, ^8^

## 3 THE SYNTHESIS AND PHYSIOLOGICAL FUNCTIONS OF CATHEPSIN B

CTSB, a member of the C1 family, is extensively expressed in various tissues and cell types throughout the body, often referred to as a housekeeping enzyme.^9^ The synthesis of CTSB begins with the formation of a 339 amino acid precursor enzyme along with a signal peptide complex consisting of 17 amino acids in the rough endoplasmic reticulum (RER). And this process leads to the generation of an inactive precursor form of CTSB. Subsequently, the precursor CTSB is transferred from the RER to the Golgi apparatus. Within the Golgi apparatus, two asparagine residues of the precursor CTSB undergo glycosylation to form phosphorylated mannose residues. The phosphorylated precursor CTSB binds to the mannose‐6‐phosphate receptor in the Golgi network and is then conveyed to the lysosomes through transport vesicles.^9^ Inside the acidic environment of the lysosomes, the precursor CTSB undergoes self‐catalytic activation through proteolytic cleavage, resulting in the formation of active CTSB. This activation process involves the cleavage of 6 amino acid residues from the C‐terminus to yield the mature single‐chain form, while hydrolysis between residues 47 and 50 produces a double‐chain form comprising a 25 kDa heavy chain and a 5 kDa light chain.^1^

The primary role of CTSB involves the degradation of non‐specific proteins that enter the lysosomal system from the extracellular space through processes such as phagocytosis or endocytosis, as well as from other intracellular compartments.^3^, ^9^ This degradation results in the release of small peptides and amino acids that permeate through the lysosomal membrane and are subsequently utilized by the cell, contributing to the maintenance of cellular homeostasis and overall survival.^10^, ^11^

Evidence from animal models and clinical studies in pathology and biomarkers supports the notion that CTSB serves non‐lysosomal roles, including participation in signalling cascades,^12^ immune regulation,^13^ degradation of extracellular matrices,^14^ cell apoptosis,^15^ autophagy,^16^ pyroptosis^17^, ^18^ and other cellular processes. Moreover, a growing body of scholars has identified the significant involvement of CTSB in cardiovascular diseases.

## 4 CATHEPSIN B IN CARDIOVASCULAR DISEASES

### 4.1 Cathepsin B and atherosclerosis

Atherosclerosis (AS) serves as the pathological basis for the majority of cardiovascular diseases. It is distinguished by extensive restructuring of the ECM within the vascular wall induced by chronic inflammation, leading to lipid deposition.^19^, ^20^ Key aspects of AS encompass the progressive accumulation of inflammatory cells and lipids.^21^, ^22^, ^23^ Research indicates elevated levels of CTSB expression in atherosclerotic lesions and circulation of both atherosclerotic animals and patients, implicating its role in the development of atherosclerotic plaques through processes including lipid metabolism, inflammation and other pathological cascades. This will be discussed in more detail in the subsequent text.

In 2002, a study^24^ revealed elevated CTSB expression in AS lesions. The application of near‐infrared imaging systems identified notably high levels of CTSB expression in apoE‐deficient or apoE/endothelial nitric oxide (NO) synthase double knockout mice, particularly in regions abundant in macrophages exhibiting pronounced inflammation, showing a significant increase in CTSB mRNA and protein levels.^25^ This finding was further supported in the atherosclerotic model of New Zealand white rabbits that underwent balloon‐denudation of iliac arteries and were fed a hypercholesterolemic diet: histopathological analysis revealed co‐localization of immunoreactive macrophages and CTSB in the plaques.^26^

Moreover, in human carotid artery plaques, based on cathepsin activity probe technology, it was found that CTSB activity increased in unstable carotid artery plaques. The activity of CTSB in M2 macrophages, which exhibit a fatty acid oxidation‐dependent phenotype and anti‐inflammatory properties,^27^ is significantly higher in unstable plaques compared to stable plaques.^28^ This finding suggests that CTSB contributes to the clinical sequelae of AS and M2 macrophages are pivotal in the development of advanced vascular lesions.

#### 4.1.1 Cathepsin B is involved in the inflammation

The adverse impact of inflammatory processes on arterial wall function is a key contributor to the formation of AS.^29^, ^30^ CTSB exacerbates the inflammatory burden of cells or tissues by modulating the activity of inflammatory cells and the synthesis of inflammatory molecules.

CTSB has been shown to exacerbate AS by promoting pyroptosis,^31^ a form of programmed cell death that is inflammatory in nature. In experiments involving ApoE‐deficient, overexpression of CTSB led to increased atherosclerotic plaque formation and enhanced pyroptosis in vascular smooth muscle cells (VSMCs) when exposed to oxidized low‐density lipoprotein (ox‐LDL).^32^ In other vitro experiments, CTSB is also implicated in the secretion of interleukin‐1β (IL‐1β) mediated by the nucleotide‐binding domain‐like receptor protein 3 (NLRP3) inflammasome.^33^ In experiments with THP‐1 macrophages treated with a pharmacological specific inhibitor CA‐074 Me, the activation of NLRP3 inflammasome induced by oxLDL is effectively suppressed, resulting in a decrease in IL‐1β release.^34^ Moreover, studies have demonstrated that CTSB is responsible for orchestrating the formation of the NLRP3 inflammasome complex triggered by palmitate in cultured microvascular endothelial cells (ECs). This activation leads to the stimulation of caspase‐1 and an increase in IL‐1β production, consequently enhancing endothelial cell permeability.^35^, ^36^ Similarly. genetic knockdown of CTSB using siRNA transfection and pharmacological inhibition of CTSB with CA‐074 Me can suppress nicotine‐induced NLRP3 inflammasome activation in VSMCs.^37^ All the aforementioned processes involve the activation of the NOD‐like receptor protein 3 (NLRP3) inflammasome, which is crucial for the inflammatory response in AS.^21^ Additionally, in macrophages, CTSB activity is involved in the post‐translational processing and production of lipopolysaccharides‐induced tumour necrosis factor‐α(TNF‐α). CTSB‐deficient and CTSB inhibition with CA‐074 Me, or with short interfering RNA leads to the inability of vesicles containing TNF‐α precursor to be transported to the plasma membrane.^38^

**FIGURE 1:**
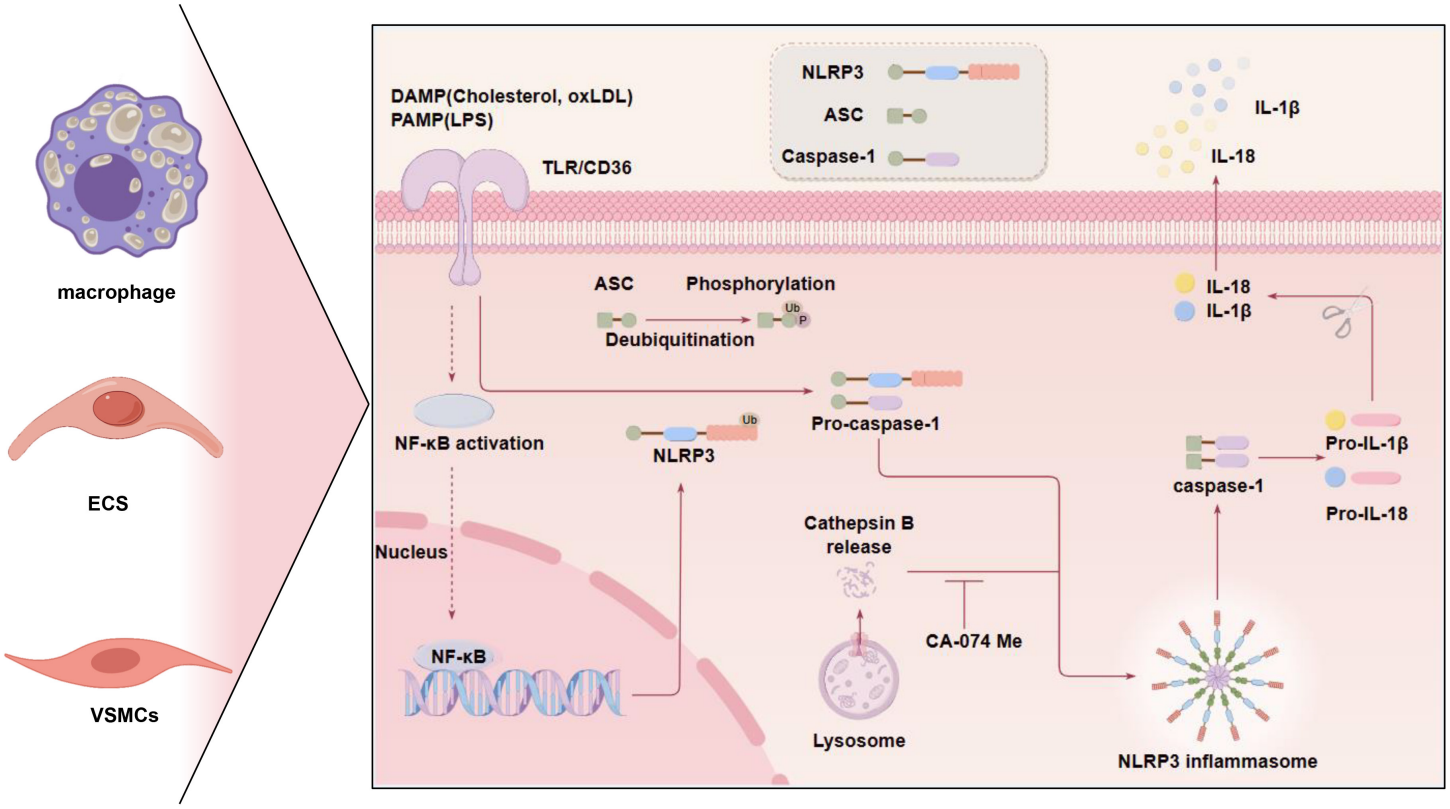
In the pathogenesis of atherosclerosis, CTSB contributes to the activation of the NLRP3 inflammasome in macrophages, ECs and VSMCs, thereby promoting the secretion of inflammatory cytokines and pyroptosis. DAMP, such as cholesterol and oxLDL, alongside PAMP like LPS, via pattern recognition receptors like TLR and CD36, triggers NF‐κB signalling and the release of cathepsin B from lysosomes, ultimately leading to the activation of the NLRP3 inflammasome and an increase in the secretion of IL‐1β and IL‐18. The administration of the CTSB inhibitor CA‐074 Me effectively inhibits NLRP3 activation, thereby mitigating inflammation. ASC, apoptosis‐associated speck‐like protein containing CARD; CA‐074 Me, a CTSB inhibitor; DAMP, damage associated molecular pattern; ECs, endothelial cells; IL‐18, interleukin‐18; IL‐1β, interleukin‐1β; LPS, lipopolysaccharides; NF‐kB, nuclear factor‐kappa B; NLRP3, NOD‐like receptor thermal protein; oxLDL, oxidized low‐density lipoprotein; PAMP, pathogen associated molecular pattern; TLR, toll‐like receptors; VSMCs, vascular smooth muscle cells. The figure was created by Figdraw.

The involvement of CTSB in the initiation and progression of inflammation in diverse cell types during atherosclerotic plaque advancement is evident (Figure 1). Inhibiting CTSB activity can ameliorate the inflammatory response of cells and impede the progression of AS.

#### 4.1.2 Cathepsin B participates in lipid metabolism

The accumulation of lipids and metabolic abnormalities plays a crucial role in the development of AS. One of the key early features of AS is the presence of lipid‐laden macrophages known as foam cells, which become trapped in the arterial walls.

Apolipoprotein A‐I (apoA‐I), the primary protein constituent of high‐density lipoprotein (HDL), plays a pivotal role in the anti‐atherogenic export of cholesterol from macrophage‐derived foam cells.^39^ Studies have shown that CTSB released by macrophages can markedly diminish the solubility of apoA‐I by cleaving it at Ser228 in the C‐terminal region, consequently impairing its cholesterol efflux capacity and worsening the progression of AS.^40^

Dysfunction of the lysosomal system in the uptake and processing of low‐density lipoproteins (LDL) is a crucial factor contributing to the lipid accumulation within macrophages in the arterial vessel wall. Diminished activity of CTSB in macrophage lysosomes leads to the buildup of modified proteins and lipids in AS.^41^ Certain oxidants like hypochlorous acid (HOCl), hypothiocyanous acid (HOSCN) and LDL modified by these oxidants can markedly reduce CTSB activity in J774A.1 mouse monocyte macrophages, resulting in lysosomal dysfunction and disruption of LDL processing and hydrolysis by the cells.^42^

As a result, CTSB is intricately linked to the pathogenesis of AS and primarily operates within macrophages. While secreted CTSB can enhance AS progression, the enzymatic activity of CTSB in lysosomes is vital for degrading modified proteins and lipids in AS. One of the pivotal questions still remains to be answered, that is, whether the upregulation of CTS in lipid metabolism exerts a protective or stimulatory influence on the development of AS. Further laboratory and clinical investigations are imperative to elucidate this issue.

#### 4.1.3 Circulating cathepsin B as prognostic and diagnostic biomarkers for ASCVDs

The literatures have underscored the potential of circulating CTSB as prognostic biomarkers for cardiovascular disease. In a 10‐year follow‐up of the CLARICOR trial, it was observed that in patients with stable coronary heart disease, serum CTSB levels were found to be associated with high risk for cardiovascular events.^43^ Subsequently, a clinical study provided evidence supporting the findings of the CLARICOL study by demonstrating a link between CTSB expression in peripheral blood mononuclear cells and arterial stiffening as well as atherosclerotic vascular disease of patients with coronary artery disease (CAD).^44^

Therefore, these findings suggest that elevated levels of CTSB could serve as promising biomarkers for atherosclerotic cardiovascular disease (ASCVD) and treatment response. Large‐scale clinical cohort studies are still necessary to ascertain the prognostic and diagnostic potential of heightened circulating levels of individual CTSB in individuals with ASCVD.

#### 4.1.4 Current optical probes for imaging cathepsin B in atherosclerosis

Numerous circulating and tissue cysteinyl cathepsins (CTSs) have attracted interest as potential therapeutic targets in individuals with ASCVD through the examination of AS using fluorescent probes, nanoparticles and radiolabeled inhibitors.^31^

The tomographic in vivo imaging method along with catheter‐based optical sensing methods suggests that CTSB could potentially serve as a biomarker for vulnerable plaques when examined with beacons.^25^, ^45^ Subsequent experimental and clinical molecular imaging studies have shown that CTSB near‐infrared fluorescence (NIRF) probes can be utilized as molecular imaging biomarkers for AS and monitor the therapeutic effects of drug applications.^46^, ^47^ Utilizing quenched fluorescent cathepsin activity‐based probes (ABPs) that target CTSB activity, it's possible to perform non‐invasive and accurate detection of atherosclerotic plaques in mice through fluorescence molecular tomography (FMT). Additionally, ex vivo fluorescence imaging can identify cathepsin activity within plaque macrophages.^48^ In addition, optical or dual‐modal probes BMV109 and BMV101 targeting CTSB were individually administered via the tail vein of atherosclerotic plaque mice for non‐invasive imaging utilizing optical and small animal PET/CT. These probes exhibited a comparable specific labeling of activated macrophage clusters and were applied for localized labeling of human carotid plaques.^49^ The research conducted by Elrahman et al. similarly revealed that targeting cathepsin activity in human carotid plaques could provide a novel diagnostic approach for identifying high‐risk plaques.^28^ Consistently, NIRF imaging with P‐ICG2‐PS‐Lip targeting CTSB could be valuable for detecting atherosclerotic plaques prone to embolism.^50^ A recently developed lipid‐unlocked CTSB‐responsive probe (L‐CRP) can non‐invasively and accurately image CTSB within lipid‐rich plaques, dynamically reporting CTSB levels within the plaques, which are closely related to plaque vulnerability features including fibrous cap thickness, macrophage recruitment and necrotic core size.^51^

Additionally, preclinical studies have demonstrated the efficacy of photodynamic therapy utilizing CSTB activatable theranostic agent (L‐SR15) or photosensitizing quenched activity‐based probes (PS‐qABP) targeting CTSB in reducing vascular inflammation and atherosclerotic plaque progression.^52^, ^53^ This means that utilizing CTSB‐mediated therapeutic diagnostic agents can enable simultaneous diagnostic visualization and therapeutic interventions. Future advancements in technologies utilizing CTSB‐specific probes hold promise for clinical applications in patients with AS. The overarching objective is to devise a more efficient non‐invasive approach for diagnosing ASCVD and predicting its prognosis.

The findings suggest the potential utility of these probes in non‐invasive imaging for promptly assessing the progression of atherosclerotic disease and the vulnerability of plaques (Table 1). Simultaneously, the synchronization of diagnosis and treatment can be achieved, offering potential assistance in diagnosing plaque vulnerability and predicting its prognosis in clinical applications.

**TABLE 1:** Summary of the current optical probes for imaging CTSB in atherosclerosis.

Author	Year	Probe	Imaging model	Application	Animal model	Ref.
Jiqiu Chen et al.	2002	CTSB–sensitive NIRF probe	Fluorescence	In vivo imaging	Mice	^25^
Dong‐Eog Kim et al.	2009	CTSB–activated NIRF probe	Fluorescence	In vivo imaging	Mice	^54^
Shu‐An Lin et al.	2012	CTSB and α(v)β (3) integrin NIRF probe	Fluorescence	In vivo imaging	Mice	^46^
Soo‐Min Shon et al.	2013	L‐SR15	Fluorescence	In vivo imaging	Mice	^53^
Ihab Abd‐Elrahman et al.	2016	GB123/GB137 ABPs	Fluorescence	In vivo imaging	Mice	^48^
Nimali P Withana et al.	2016	BMV109 and BMV101	Fluorescence	In vivo and ex vivo imaging	Mice and human	^49^
Ihab Abd‐Elrahman et al.	2016	GB123	Fluorescence	Ex vivo imaging	Human	^28^
Marcella A Calfon Press et al.	2017	Prosense VM110	Fluorescence	In vivo and ex vivo imaging	Rabbit	^47^
Yudai Narita et al.	2019	P‐ICG2‐PSLip	Fluorescence	In vivo and ex vivo imaging	Mice and rabbits	^50^
Tommy Weiss‐Sadan et al.	2019	PS‐qABP	Fluorescence	In vivo and ex vivo imaging	Mice and human	^52^
Yuan Ma et al.	2023	L‐CRP	Photoacoustic	In vivo and ex vivo imaging	Mice and human	^51^

### 4.2 Cathepsin B and myocardial infarction

The vulnerability and rupture of atherosclerotic plaques in the coronary arteries are critical determinants for myocardial infarction (MI). Characteristics of vulnerable plaques include a thin fibrous cap, reduced smooth muscle cell (SMC) count, a necrotic core abundant in cholesterol crystals and notable infiltration of inflammatory cells.^55^ Early research reported a substantial increase in CTSB expression within atherosclerotic lesions.^24^ CTSB is predominantly expressed in cells surrounding the lipid core of the plaques, primarily foam cells derived from macrophages.^24^ It plays a pivotal role in plaque inflammation, serving as a potential biomarker for vulnerable plaques^25^, ^54^ and identifying individuals at high risk of acute MI.^56^ Three weeks post‐MI, the expression of CTSB markedly escalates in monocytes isolated from atherosclerotic plaque tissues,^57^ suggesting a potential involvement of CTSB in plaque destabilization and rupture.

Subsequent studies examined the circulating levels of CTSB and cystatin C, a cysteine protease inhibitor, in patients with acute MI. The research revealed an elevation in CTSB levels during the acute phase of MI, alongside a reduction in cystatin C levels.^56^ In a long‐term follow‐up subclinical trial known as CLARICOR^43^ (effect of clarithromycin versus placebo on mortality and morbidity in patients with ischaemic heart disease), serum CTSB levels were assessed in 4372 stable coronary heart disease patients. The long‐term follow‐up results indicated that elevated circulating CTSB levels were associated with an increased risk of cardiovascular events, suggesting its potential utility as a risk marker for predicting adverse cardiovascular outcomes in patients with stable coronary heart disease. A recent study found that plasma CTSB levels increased in MI patients after percutaneous coronary intervention (PCI) compared to healthy controls, and were positively correlated with plasma cTnI levels, a biomarker for myocardial injury. Furthermore, the elevation of CTSB persisted up to 24 h post‐PCI.^58^

Numerous studies in the field of animal experimental research have demonstrated a significant upregulation of CTSB expression in the serum and heart of rats with isoproterenol (ISO)‐induced MI.^59^, ^60^, ^61^ This upregulation of CTSB can activate downstream pro‐inflammatory mediators, thereby playing a role in the pathophysiological processes of MI.^61^ Additionally, the CTSB inhibition has been shown to effectively mitigate cardiac dysfunction, limit infarct size and reduce inflammatory invasion in Sprague–Dawley rats following left anterior descending coronary artery ligation, achieved through the inhibition of NLRP3 activation.^58^

From the information presented above, it can be inferred that CTSB significantly impacts MI, potentially participating in critical pathological mechanisms like plaque rupture and post‐MI myocardial remodelling. Additionally, the circulating levels of CTSB are correlated with the severity of CAD in patients and the incidence of adverse cardiovascular events.

### 4.3 Cathepsin B and hypertension

While research on CTSB in hypertension has a long history, the specific mechanisms are still unclear. Early studies revealed elevated CTSB enzyme activity in the aortic ECs of aged spontaneously hypertensive rats (SHR) and rats with renal hypertension resulting from ligation of the posterior branches of both renal arteries in male Wistar rats of the Kyoto strain, with this activity showing an age‐dependent increase.^62^ Furthermore, in mice with renal hypertension induced by NPHS2 gene knockout, western blot and immunohistochemical staining analyses revealed a notable increase in CTSB expression in podocytes, glomeruli and intercalated cells.^63^ In hypertensive cardiomyopathy induced by aortic ligation, CTSB expression in the heart is significantly upregulated,^64^ promoting myocardial remodelling through the regulation of the TNF‐α/ASK1/JNK signalling pathway. Additionally, CTSB knockout markedly alleviates pressure overload‐induced cardiomyocyte hypertrophy, myocardial fibrosis, cell apoptosis and cardiac dysfunction. Recently, a study identified significant associations between circulating levels of CTSB and blood pressure (BP) using various Mendelian randomization analysis techniques, which were subsequently validated with independent datasets.^65^

Renin is the initiating enzyme of the renin‐angiotensin‐aldosterone system (RAAS), playing an important role in regulating BP and water‐electrolyte balance. Researchers have found that CTSB, colocalized with renin in juxtaglomerular cell secretory granules, can cleave and activate prorenin to become catalytically active renin.^66^ Subsequent studies revealed no significant differences in plasma renin activity and mean arterial BP between CTSB gene knockout mice and wild‐type mice. The pharmacological inhibition of CTSB did not affect renal or plasma renin activity in SHR.^67^ Therefore, while CTSB demonstrates renin cleavage and activation in vitro, its mechanism of action on the RAAS system in vivo is still worth exploring. Some researchers have suggested that CTSB triggers the proteolytic cleavage of the α‐epithelial sodium channels(αENaC) protein, leading to inappropriate activation of ENaC. The application of CA‐074Me, a membrane‐permeable inhibitor of CTSB, can effectively prevent hypertension.^63^

In conclusion, the pathogenesis of CTSB in hypertension and related diseases is intricate and involves multiple cells and organs, making it complex and multifaceted. This complexity adds challenges to in vivo experimental design. Further experimental investigations are required to clarify the pathological mechanisms of CTSB in hypertension and related conditions.

### 4.4 Cathepsin B and myocarditis

Myocarditis is a nonspecific inflammatory heart disease primarily caused by viral infections.^68^ A recent study conducted by Wang et al.^18^ revealed an upregulation of CTSB expression in the hearts of mice with acute and chronic myocarditis induced by Coxsackievirus B3 (CVB3) infection, along with inflammasome activation. The suppression of CTSB significantly mitigated inflammasome activation, reduced caspase‐1‐induced cell pyroptosis, minimized inflammation and cell damage in the heart and enhanced cardiac function affected by myocarditis, ultimately increasing the survival rate in mice. Conversely, the knockout of cystatin C escalated CTSB expression, boosted inflammasome activation, intensified myocardial cell pyroptosis and exacerbated myocarditis. Therefore, CTSB can enhance cell pyroptosis by triggering inflammasomes and exacerbate CVB3‐induced myocarditis, suggesting that CTSB inhibitors could offer a promising avenue for the treatment of CVB3‐induced myocarditis.

The 2020 coronavirus disease‐2019 (COVID‐19) pandemic caused by the severe acute respiratory syndrome coronavirus 2 (SARS‐CoV‐2) often presents with a range of systemic complications.^69^ Myocardial involvement is a significant pathophysiological component in some critically ill patients with CoV‐2 infection, possibly due to myocarditis.^70^ CTSB was found to cleave the SARS‐CoV‐2 spike and be one of the necessary accessory proteins for facilitating the SARS‐CoV‐2 interaction and allow viral entry.^71^, ^72^, ^73^ The analysis of RNA expression atlases in the human embryonic heart at single‐cell resolution revealed an enrichment of CTSB in cardiomyocytes.^74^ Atrial and ventricular cardiomyocytes are possibly susceptible to SARS‐CoV‐2 infection by involving the CTSB thus leading to cardiac manifestations.^75^

### 4.5 Cathepsin B and chemotherapy‐induced cardiotoxicity

The rising prevalence of tumours has led to a growing prevalence in the development and utilization of chemotherapy medications. Doxorubicin is currently one of the most effective chemotherapy drugs, suitable for various malignant tumours, including breast cancer, lung cancer, ovarian cancer and so on. However, its adverse effects on the heart greatly limit its clinical use.^76^

Bao et al.^77^ employed doxorubicin to induce myocardial injury in H9c2 cardiomyocytes. Utilizing iTRAQ proteomic analysis and western blot techniques, they confirmed the elevated expression of CTSB in doxorubicin‐treated H9c2 cardiomyocytes. Our research findings align with this observation, indicating that co‐incubating H9c2 cardiomyocytes with DOX can induce CSTB expression.^78^ Subsequently, Moreira et al.^79^ discovered that CTSB could cleave the apoptosis‐inducing factor (AIF) before its release from mitochondria, facilitating its nuclear translocation, inducing extensive DNA fragmentation and exacerbating subsequent apoptotic responses. CTSB also has been identified to aggravate doxorubicin‐induced myocardial injury via the regulation of NF‐κB signalling.^78^ It is evident that CTSB plays a crucial role in doxorubicin‐induced cardiotoxicity.

Nilotinib is a second‐generation BCR‐ABL tyrosine kinase inhibitor and is widely used for the treatment of chronic myeloid leukaemia (CML).^80^ Cardiotoxicity is a major adverse effect associated with nilotinib therapy in CML patients. Despite its widespread use, the precise molecular mechanisms underlying this cardiotoxicity remain unclear, impeding its optimal application. In a study by Yang et al.,^81^ it was observed that treatment with nilotinib led to a notable increase in the activity of CTSB and autophagy in H9c2 cells. This heightened activity resulted in the translocation of AIF to the nucleus from the mitochondria, upregulation of p53 expression and ultimately, the induction of apoptosis. Notably, inhibition of autophagy effectively reduced CTSB activity and mitigated the cardiotoxic effects induced by nilotinib. These findings highlight the role of CTSB in the cardiotoxicity associated with various chemotherapeutic agents, primarily through its involvement in autophagy and apoptosis pathways.

### 4.6 Cathepsin B and heart failure

Heart failure (HF) represents the advanced stage of various heart diseases. As early as 2006, researchers identified a significant increase in both the protein and mRNA expression levels of CTSB in the cardiac tissue of HF patients compared to individuals without HF.^82^ Moreover, in patients diagnosed with dilated cardiomyopathy, the expression of CTSB showed a negative correlation with the ejection fraction (EF), with those exhibiting elevated CTSB levels also demonstrating heightened myocardial apoptosis.^82^ A recent study revealed a positive association between the circulating levels of CTSB and the severity of left ventricular dysfunction in dilated cardiomyopathy patients.^83^

Hence, the expression levels of CTSB in both cardiac tissue and circulation may play a crucial role in the onset and progression of HF. Nonetheless, the precise underlying mechanisms remain incompletely elucidated.

### 4.7 Cathepsin B and abdominal aortic aneurysm

Abdominal aortic aneurysm (AAA) is characterized by a localized dilation of the aorta resulting from inflammatory cell infiltration, SMC depletion and degradation of collagen and elastic proteins in the aortic vessel wall, leading to compromised vascular wall integrity.^84^

Enzyme‐linked immunosorbent assay and specific fluorescent substrate assay revealed an increase in protein expression and activity of CTSB in the aortic wall of AAA patients.^85^ Histopathological analysis of cellular localization showed that CTSB is expressed in ECs, SMC, lymphocytes and especially in macrophages.^86^ Disturbances arising from the imbalance between endogenous inhibitors regulating cysteine cathepsins have a detrimental impact on the maintenance of the aortic wall structure. This imbalance not only promotes aortic remodelling but also plays a significant role in the development of AAA.^87^ Among these proteases, CTSB plays a significant role in the degradation of ECM proteins, causing alterations in the distribution of elastic fibres and collagen within the local arterial wall.^86^ Consequently, the arterial wall loses its ability to withstand the hemodynamic forces and progressively enlarges. Therefore, regulating the expression levels of tissue proteases may provide new treatment options for AAA.

### 4.8 Cathepsin B and mucopolysaccharidosis type I

Mucopolysaccharidosis type I (MPS I) is a metabolic disorder of glycosaminoglycans resulting from lysosomal dysfunction. It is marked by the abnormal buildup of undegraded glycosaminoglycans (GAGs) due to the deficiency of α‐L‐iduronidase.^88^ This condition notably affects the cardiovascular system, characterized by myocardial hypertrophy, cardiac valve dysfunction, aortic rigidity and dilation.^89^ The exact pathogenesis of this disease is still not completely elucidated.

Research has found that GAG accumulated in the myocardial tissue of 6‐month‐old MPS I mice can recruit infiltrating macrophages, leading to the secretion of a large amount of CTSB that degrades the ECM.^90^ This process results in reduced collagen content in the MPS I cardiac valves, exacerbating valve dysfunction. Scholars have also discovered that CTSB released from lysosomes in MPS I human fibroblasts may contribute to the breakdown of ECM components in the aorta, myocardium and cardiac valves.^91^ Treatment of MPS I mice with an intravenously injected CTSB inhibitor for 4 months significantly improved indicators such as aortic dilation, thickening of cardiac valves and cardiac functional parameters.^91^ It is evident that lysosomal dysfunction in MPS I can impact the release and secretion of CTSB, influencing the pathophysiological progression of cardiovascular lesions in MPS I. Targeting CTSB could present a novel avenue for future therapeutic interventions in MPS I‐related cardiovascular complications.

## 5 CONCLUSION AND PERSPECTIVE

**FIGURE 2:**
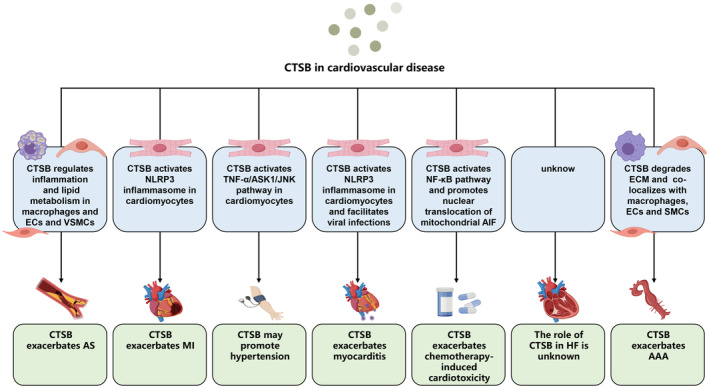
A summary of CTSB function in cardiovascular diseases. CTSB participates in the pathogenesis of various cardiovascular diseases, with a particular emphasis on its role in atherosclerosis. CTSB facilitates the activation of the NLRP3 inflammasome, contributing to the pro‐inflammatory response observed in diverse cardiovascular pathologies, including myocardial infarction, myocarditis and chemotherapy‐induced cardiotoxicity. It modulates potential signalling pathway that contributes to the progression of hypertension. In abdominal aortic aneurysm, CTSB primarily exacerbates the disease progression by degrading the extracellular matrix. However, the role of CTSB in heart failure remains unknown. AAA, abdominal aortic aneurysm; AIF, apoptosis inducing factor; AS, atherosclerosis; ASK1, apoptosis signal regulating kinase 1; CTSB, cathepsin B; ECM, extracellular matrix; ECs, endothelial cells; HF, heart failure; JNK, C‐Jun N‐terminal kinase; MI, myocardial infarction; NLRP3, NOD‐like receptor thermal protein domain associated protein 3; TNF‐α, tumour necrosis factor‐α; VSMCs, vascular smooth muscle cells.

Initially, CTSB was primarily recognized as a protease responsible for lysosomal protein hydrolysis. Nevertheless, recent research has revealed that beyond its protease function. Numerous clinical investigations and animal studies have revealed significant changes in the expression or activity of CTSB across various cardiovascular conditions (Table 2). Presently, its involvement in AS represents a focal point of investigation, particularly due to its significant predictive value in identifying unstable atherosclerotic plaques, thereby offering insights into MI prediction. CTSB also participates in the pathophysiological cascades of several other cardiovascular disorders, including HF, myocarditis, hypertensive heart disease and chemotherapy‐induced cardiotoxicity (Figure 2). Nonetheless, our research on its pathological mechanisms in cardiovascular diseases is still in its infancy, underscoring the imperative need for further in‐depth research to elucidate and delineate its specific mechanisms of action in distinct cardiovascular diseases, aiming to furnish enhanced theoretical foundations and novel perspectives for the management and prevention of clinically pertinent cardiovascular conditions.

**TABLE 2:** The expression or activity change of CTSB in cardiovascular disease.

Disease	Pathological model	Expression or activity	Site	Cell type	Ref.
Atherosclerosis	Apolipoprotein E‐deficient (ApoE−/−) mice	Increase	Lesions	Macrophages	^24^
	Western‐type diet‐fed apoE and apoE/endothelial NO synthase double knockout mice	Increase	Lesions	Macrophages	^25^
	New Zealand white rabbits underwent balloon‐denudation of iliac arteries and hypercholsterolemic diet	Increase	Lesions	Macrophages	^26^
	Human	Increase	Carotid artery plaques	Macrophages	^28^
Coronary artery disease (CAD)	Human	Increase	Peripheral blood	Mononuclear cells	^44^
Myocardial infarction (MI)	Left anterior descending coronary artery occlusion in mice	Decrease	The ischaemic area of the myocardium	‐	^92^
	Isoproterenol (ISO)‐induced MI in male Wistar rats	Increase	Serum and the heart	‐	59, 60, 61
Acute myocardial infarction (AMI)	Human	Increase	Serum	‐	^56^
Post‐myocardial infarction	Apolipoprotein E‐deficient (ApoE−/−) mice	Increase	Lesions	Ly‐6 Chigh monocytes	^57^
Myocardial ischemia/reperfusion injury	Myocardial infarction patient underwent percutaneous coronary intervention (PCI)	Increase	Plasma	‐	^58^
	Left anterior descending coronary artery ligation for 30 min and 24 h of reperfusion	Increase	Plasma and heart	Cardiomyocytes	^58^
Hypertension	Spontaneously and renal hypertensive rats	Increase	Aorta	Endothelial cells	^62^
	Renal hypertension induced by NPHS2 gene knockout	Increase	Kidney	Glomeruli and intercalated cells	^63^
	Aortic banding‐induced hypertension	Increase	Heart	Cardiomyocytes	^64^
Myocarditis	coxsackievirus B3 (CVB3) jnfected viral myocarditis (VMC)	Increase	Heart	‐	^18^
Chemotherapy‐induced cardiotoxicity	doxorubicin induced myocardial injury	Increase	‐	H9c2 cardiomyocytes	77, 78, 79
	nilotinib induced myocardial injury	Increase	‐	H9c2 cardiomyocytes	^81^
Heart failure	dilated cardiomyopathy	Increase	Left ventricles	‐	^82^
	dilated cardiomyopathy	Increase	Peripheral blood	Mononuclear cells	^83^
Abdominal aortic aneurysm(AAA)	human	Increase	Aortic wall	Endothelial cells, smooth muscle cells, macrophages	85, 86
Diabetic cardiomyopathy	streptozotocin injection	Increase	Heart	Cardiomyocytes	^93^

## 6 OUTSTANDING QUESTIONS

Further exploration of the crucial roles of CTSB and its application in the treatment of cardiovascular diseases requires significant optimization, including:

1. Further large‐scale cohort studies are necessary to confirm the predictive value of CTSB in assessing cardiovascular risk among patients with AS.
2. Future research will focus on further exploring the safety profile of CTSB‐based probes to establish a basis for their clinical utilization.
3. An elevation in CTSB levels has been noted in HF; however, given the diverse aetiology of HF, is this expression uniform across all HF types, including HF with reduced EF (HFrEF) and HF with preserved EF (HFpEF)? If so, what accounts for the variations in their expression, and does CTSB play a role in the pathogenesis of HF?
4. While alterations in CTSB expression or activity have been identified in numerous cardiovascular diseases, what are the underlying factors driving these changes?
5. Research studies are required to explore the utilization of small molecule CTSB inhibitors in cardiovascular disorders characterized by elevated CTSB expression.

## AUTHOR CONTRIBUTIONS

**Zhulan Cai:** Conceptualization (equal); writing – original draft (equal). **Shunyao Xu:** Conceptualization (equal); **Chen Liu:** Conceptualization (equal).

## CONFLICT OF INTEREST STATEMENT

The authors declare no conflicts of interests.

## Data Availability

Data sharing is not applicable to this article as no new data were created or analysed in this study.
